# Anoikis resistance in diffuse glioma: The potential therapeutic targets in the future

**DOI:** 10.3389/fonc.2022.976557

**Published:** 2022-08-15

**Authors:** Zhengyang Zhu, Chaoyou Fang, Houshi Xu, Ling Yuan, Yichao Du, Yunjia Ni, Yuanzhi Xu, Anwen Shao, Anke Zhang, Meiqing Lou

**Affiliations:** ^1^ Department of Neurosurgery, Shanghai General Hospital, Shanghai Jiao Tong University School of Medicine, Shanghai, China; ^2^ Department of Neurosurgery, Huashan Hospital, Shanghai Medical College, Fudan University, Shanghai, China; ^3^ Department of Neurosurgery, Second Affiliated Hospital, School of Medicine, Zhejiang University, Hangzhou, China; ^4^ Department of Neurosurgery, Clinical Research Center for Neurological Diseases of Zhejiang Province, Hangzhou, China

**Keywords:** anoikis resistance, glioma, molecular pathways, therapeutic targets, apoptosis

## Abstract

Glioma is the most common malignant intracranial tumor and exhibits diffuse metastasis and a high recurrence rate. The invasive property of glioma results from cell detachment. Anoikis is a special form of apoptosis that is activated upon cell detachment. Resistance to anoikis has proven to be a protumor factor. Therefore, it is suggested that anoikis resistance commonly occurs in glioma and promotes diffuse invasion. Several factors, such as integrin, E-cadherin, EGFR, IGFR, Trk, TGF-β, the Hippo pathway, NF-κB, eEF-2 kinase, MOB2, hypoxia, acidosis, ROS, Hsp and protective autophagy, have been shown to induce anoikis resistance in glioma. In our present review, we aim to summarize the underlying mechanism of resistance and the therapeutic potential of these molecules.

## Introduction

Glioma is the most common central nervous system (CNS) tumor and has limited therapeutic options and poor overall survival (OS) (1, 2). Glioma comprises approximately 30% of all brain tumors and 80% of all malignant brain tumors in adults (3). According to the 5^th^ edition of the WHO classification of CNS tumors, gliomas are divided into 4 different families (adult-type diffuse gliomas, pediatric-type diffuse low-grade gliomas, pediatric-type diffuse high-grade gliomas, circumscribed astrocytic gliomas) and 17 subfamilies (4). High-grade gliomas tend to infiltrate diffusely, causing extensive areas of necrosis and hypoxia. Furthermore, the diffusive property makes it almost impossible to remove gliomas completely through surgery, leading to frequent recurrence and eventually death. Apart from surgery, radiotherapy (RT) and chemotherapy are also applied in clinical practice. However, high-precision conformal RT, temozolomide (TMZ) and bevacizumab fail to improve OS (5).

The diffusive property of glioma relates to aberrant changes in cell adhesion (6). This suggestion links glioma with a type of programmed cell death called anoikis. Anoikis is a special form of apoptosis activated upon cell detachment. Normal cells live in a microenvironment that is composed of extracellular matrix and various supporting cells. This three-dimensional scaffold provides adherent cells with necessary biochemical and mechanical signals for survival, growth, differentiation and other physiological processes (7). Remodeling of the microenvironment or cell detachment can lead to the activation of anoikis and eventually cell death.

Anoikis is regulated by many factors and pathways in wide range of cancers (8–10). By utilizing these factors, we might inhibit the diffuse infiltration of glioma, thus improving clinical outcomes. Studies have revealed that several regulators take part in anoikis resistance in glioma; these include integrin, E-cadherin, EGFR, IGFR, Trk, TGF-β, the Hippo pathway, NF-κB, eEF-2 kinase, MOB2, hypoxia, acidosis, reactive oxygen species (ROS), heat shock proteins (Hsps) and protective autophagy. Here, we review these regulators that have been reported to induce anoikis resistance in diffuse glioma and their possible utilization in future treatment.

## Downstream mechanism of anoikis

In terms of downstream mechanisms, anoikis is similar to apoptosis, which generally can be converted into the intrinsic pathway and the extrinsic pathway and converges at the activation of Caspases. The intrinsic pathway relies on mitochondrial permeabilization, which can be triggered by cellular signals such as DNA damage and unfolded protein response, followed by Caspase activation (11, 12). Mitochondrial permeabilization is a result of membrane pore formation or mitochondrial swelling. The Bcl-2 protein family controls the formation of pores in the outer mitochondrial membrane (13). In other words, the relative ratio of prosurvival (Bcl-2, BCL-xL) and proapoptotic (Bax, Bak and BH3-only subfamily) proteins of this family determines whether permeabilization occurs. During this process, Bax and Bak translocate to the outer mitochondrial membrane and create pores through oligomerization. BH3-only proteins act as activators and sensitizers. Activators promote the formation of Bax-Bak oligomers, and sensitizers act as competitive inhibitors of Bcl-2, thus neutralizing the inhibitory effect of Bcl-2 on activators and oligomers (14, 15). Permeabilization of mitochondria allows the leakage of several apoptotic proteins, such as cytochrome c (Cyt c), endonuclease G (EndoG), apoptosis-inducing factors (AIFs), second mitochondrial activator of Caspases (Smac) and high temperature requirement protein A2 (HtrA2). Cyt c is the main contributor to the intrinsic pathway, and its leakage leads to the formation of apoptosomes with apoptotic protease activating factor-1 (Apaf-1), thereby activating Caspase-9. EndoG and AIF participate in Caspase-independent DNA fragmentation. Smac and HtrA2 activate Caspase by neutralizing the apoptosis inhibitor IAP. These proteins eventually lead to anoikis *via* different mechanisms (14).

The extrinsic pathway relies on the ligand binding of death receptors on the cell membrane following the formation of the death-inducing signaling complex (DISC). Tumor necrosis factor receptor (TNFR) subfamily members, such as the first apoptosis signal (Fas) receptor, TNFR1, TNFR2 and TNF-related apoptosis inducing ligand (TRAIL) receptor-1 and -2, are major initiators of this pathway (15, 16). The ligand binding of FAS receptor results in the formation of the death-inducing signaling complex (DISC). DISC recruits and activates Caspase-8 through interaction with Fas-associated death domain protein (FADD). Activated Caspase-8 in turn activates the effectors Caspase-3, Caspase-6, and Caspase-7 (15, 17). TRAIL receptor activates Caspase-8 in a similar way as Fas receptor—via the formation of the Fas/TRAIL DISC (18). TNFR has a more complex way of inducing anoikis. First, upon ligand binding, TNFR1 binds to TNFR1-associated death domain protein (TRADD), while TNFR2 binds to TNFR-associated factor 1 (TRAF1) and TRAF2. The TNFR1-TRADD oligomer then recruits TRAF2 or TRAF5 and cellular inhibitor of apoptosis protein 1 (cIAP1) or cIAP2 to form TNFR1 complex I. Complex I further recruits the linear ubiquitin chain assembly complex (LUBAC) and ubiquitylates receptor-interacting serine/threonine-protein kinase 1 (RIPK1). Then, according to the ubiquitylation status of RIPK1, TNFR1 complex IIa or IIb is formed with RIPK1, FADD, pro-Caspase-8, the long isoform of FLICE-like inhibitory protein (FLIP_L_) and TRADD (in IIa) or RIPK3 (in IIb). Both TNFR1 complex IIa and IIb are capable of Caspase-8 activation (19).

Despite the differences between the intrinsic and extrinsic pathways, they merge at the point of Caspase activation and are connected by several shared intermediates. For example, Caspase-8, the key downstream molecule of the extrinsic pathway, also activates Bid, which is a BH3-only protein, therefore triggering the intrinsic pathway. Mitochondrial permeabilization is often followed by death receptor activation (15). Thus, anoikis is regulated by both pathways.

## Adhesion molecules and anoikis resistance

**Figure 1:**
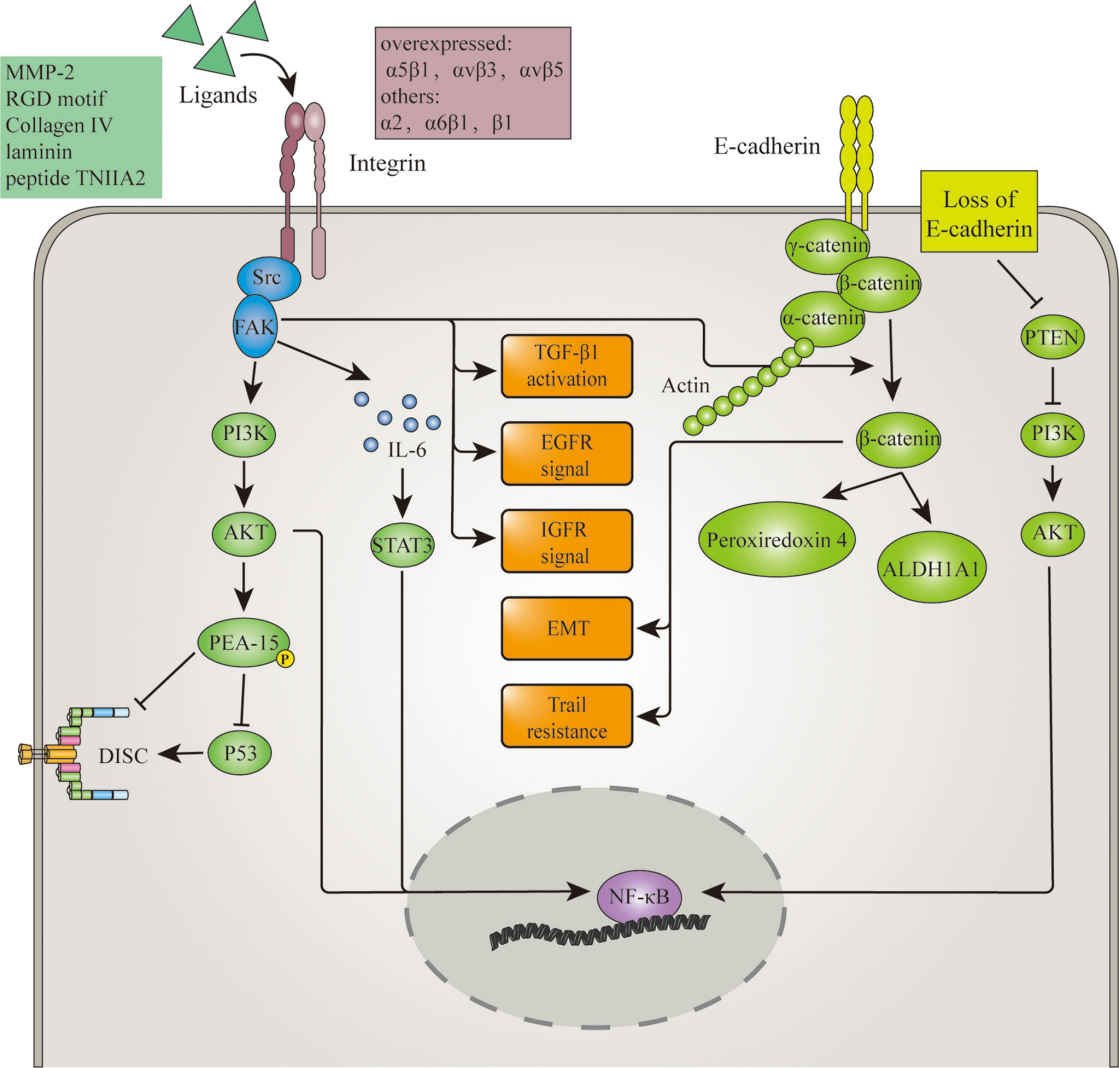
Adhesion molecules involved in anoikis resistance mechanism of glioma. Overexpression of Integrins and loss of E-cadherin in glioma disrupt the initiating signal of anoikis. Upon cell detachment, overexpressed integrins still activate FAK, which in turn activates signalings such as TGFβ, EGFR, IGFR, PI3k/AKT pathway. Then through p53 inhibition, NF-κB activation or other mediators, these pathways lead to anoikis. α2 integrin induced FAK activation also promotes the release of β-catenin from the E-cadherin/β-catenin complex. Along with the loss of E-cadherin and the help of Wnt, this increased the cytoplasmic level of β-catenin. β-catenin thereby promote anoikis resistance through EMT, TRAIL resistance and upregulation of invasion-related protein ALDH1A1 and peroxiredoxin 4. Aside from β-catenin, loss of E-cadherin also downregulates tumor suppressor PTEN and therefore maintains NF-κB activation.

Adhesion molecules are first-line mediators in anoikis signaling. They are directly involved in the establishment of cell adhesion and therefore can convey both mechanical and molecular signals of cell detachment. Thus, anomalies in adhesion molecule expression or signaling could result in anoikis resistance. Adhesion molecules such as integrins and E-cadherin have been proven to regulate anoikis resistance in glioma. And their regulation of anoikis resistance is demonstrated in **Figure 1**.

### Integrins

Integrins are transmembrane receptors that facilitate cell–cell and cell-extracellular matrix (ECM) adhesion. They bind fibronectin, vitronectin, collagen, tenascin and laminin, which are directly related to adhesion. Upon ligand binding, integrins activate the receptor tyrosine kinase (RTK) signaling pathway and thereby regulate cellular events such as growth, division, survival, differentiation and programmed death (20, 21). Loss of adhesion can trigger anoikis through this pathway. However, aberrations in the expression of integrins and their ligands often inhibit anoikis during cell detachment.

Integrins are obligate heterodimers composed of α and β subunits. There are 24 integrins in total, and a single cell generally has multiple types of integrins on its surface (21). The α5β1, αvβ3 and αvβ5 integrins are overexpressed in glioma (22). Their binding with the tripeptide sequence Arg-Gly-Asp (RGD) motif in ECM proteins activates the cytosolic tyrosine kinase Src. Src constitutively binds to the integrin β cytoplasmic tail and focal adhesion kinase (FAK) (23). In turn, FAK activation induces anoikis inhibition by activating the transcription factor NF-κB through the PI3K/AKT signaling pathway (24). This inhibition can be reversed by applying the RGD-integrin antagonist 1a-RGD, which downregulates the phosphorylation of FAK (25, 26). The binding of α5β1 integrin with matrix metalloproteinase-2 (MMP-2), which breaks down ECM and induces metastasis, promotes IL-6/Stat3 survival signaling and upregulates NF-κB, leading to anoikis resistance (27). The upregulated binding of α5β1 integrin also inhibits p53-induced apoptosis through the PI3K/AKT pathway, which activates the antiapoptotic function of astrocytic phosphoprotein PEA-15 (28). Moreover, increased binding of MMP-2 with αvβ3, which is upregulated by p21-activated kinase 4 (PAK4), activates the EGFR pathway and induces anoikis resistance (29). αV integrin also participates in TGF-β1 activation, which promotes anoikis resistance in detached glioma cells (30–32). The binding of α6β1 integrin with laminin also enhances glioma proliferation and anoikis resistance, although the involved signaling pathway is not known (33). Similarly, an *in vivo* study demonstrated the promoting role of the binding of β1-integrin with tenascin-C-derived peptide TNIIA2 in anoikis resistance in GBM without a clear understanding of its subsequent signaling (34).

### E-cadherin

E-cadherin is a type of calcium-dependent cell adhesion molecule that is important in the formation of adherens junctions to allow cells to adhere to each other. E-cadherin is also referred to as the “suppressor of invasion” because its repression is the major event responsible for dysfunction in cell–cell adhesion. Cell–cell adhesion is achieved through the formation of homodimers in membranes and the binding of the protein complex to the cytoplasmic portion of E-cadherin. E-cadherin anchors to the cytoskeleton *via* a complex composed of α-, β- and γ-catenin (35). β-Catenin is released upon E-cadherin tension relaxation due to Src-dependent FAK activation, which is integrin related. Then, with the help of Wnt, cytoplasmic β-catenin accumulates and translocates to the nucleus (36–38). Therefore, E-cadherin activates β-catenin upon cell detachment.

A previous study showed that β-catenin can inhibit anoikis resulting from the loss of cell-substrate contact (39, 40). It has also been revealed that ALDH1A1 and peroxiredoxin 4 are direct targets of β-catenin and contribute to glioma invasiveness (41–43). Thus, the E-cadherin/β-catenin/ALDH1A1 and E-cadherin/β-catenin/peroxiredoxin-4 pathways are likely direct promoters of anoikis resistance in glioma.

Furthermore, loss of the E-cadherin/β-catenin complex inhibits anoikis through epithelial-to-mesenchymal transition (EMT). EMT is a process by which epithelial cells lose their cell polarity and cell–cell adhesion and gain migratory and invasive properties to become mesenchymal stem cells (44). Loss of cell–cell contact or E-cadherin upregulates cytoplasmic β-catenin, which, together with transforming growth factor-β (TGF-β), triggers EMT. During EMT, adherens junctions are downregulated, and anoikis is inhibited (38). TRAIL sensitivity is downregulated in this process, which partially explains the common occurrence of TRAIL resistance in glioblastoma (GBM) (45, 46).

Another target protein of E-cadherin is PTEN, a tumor suppressor antagonizing the PI3K-AKT signaling pathway (47). E-cadherin upregulates PTEN expression, which inhibits NF-κB activation through the PI3K/AKT pathway, thus promoting anoikis. In contrast, PTEN mutation in GBM leads to constitutive activation of AKT signaling and anoikis resistance (48). Therefore, loss of E-cadherin promotes anoikis resistance through the downregulation of PTEN.

## Signaling and anoikis resistance in glioma

**Figure 2:**
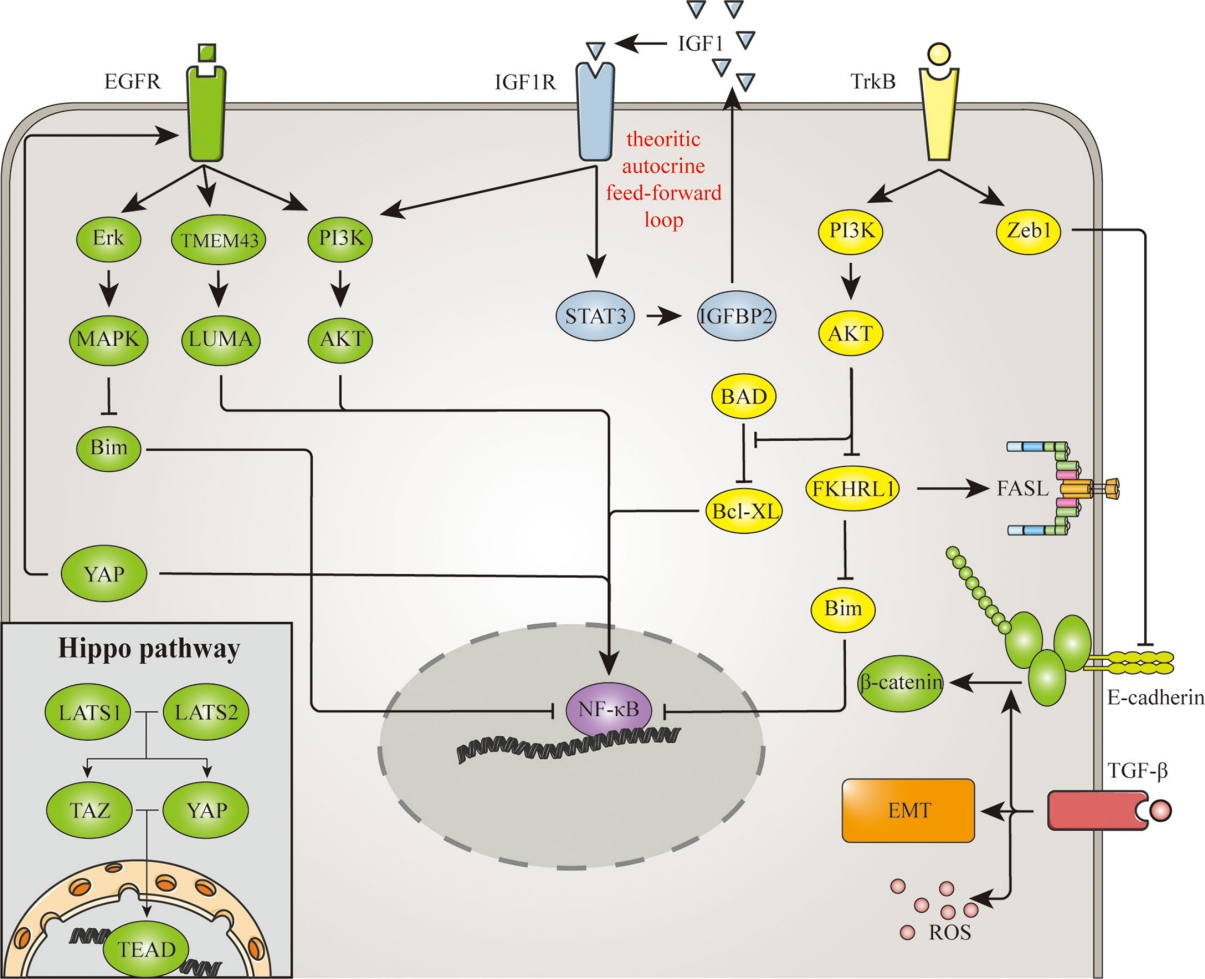
Signaling involved in anoikis resistance mechanism of glioma. EGFR, IGFR, TrkB, TGF-β and the hippo pathway are signaling pathways involved in the anoikis resistance of glioma. NF-κB is a common mediator in these pathways, for it regulates the expression of several anoikis-related proteins. Similarly, the hippo pathway promotes anoikis resistance through its transcription factor TAZ. Apart from transcription factors, EGFR also represses Bim expression through Erk/MAPK pathway. Meanwhile TrkB upregulates Bcl-xl and downregulates FasL and Bim through PI3K/AKT pathway. The signaling of TrkB and TGF-β triggers EMT as well, which promotes cell detachment and anoikis resistance. And there are also EGFR signaling triggered by the hippo pathway, and β-catenin promoted by TGF-β, indicating the connection between adhesion molecules and signaling pathways goes both way.

Cell detachment also affects extracellular signaling molecules and membrane receptors, thereby inducing anoikis through various signaling pathways. On the other hand, aberration in signaling interrupts the anoikis process that normally occurs after cell detachment. Among these signaling pathways, epidermal growth factor receptor (EGFR), insulin-like growth factor receptor (IGFR), tropomyosin receptor kinases (Trk), transforming growth factor-β (TGF-β) and the Hippo pathway have been shown to regulate anoikis resistance in glioma. And these signaling are demonstrated in **Figure 2**.

### EGFR

EGFR is a transmembrane receptor tyrosine kinase for the epidermal growth factor (EGF) family (49). Known ligands of EGFR include EGF, TGF, AREG, EPGN, BTC, EREG and HBEGF. Numerous studies have shown that EGFR has an important role in cancer progression. Its major downstream signaling pathways include the PI3K/AKT pathway, MAPK pathway, PLCγ/PKC and JAK2/STAT3 pathway (50–52). Through these pathways, EGFR can regulate anoikis resistance in gliomas. Furthermore, amplified EGFR was found in 60% of GBMs (53).

Under physiological conditions, Bim, one of the BH3-only proteins, is upregulated *via* downregulation of EGFR signaling upon cell detachment and then triggers the intrinsic pathway of anoikis. *In vitro* experiments suggest that overexpression of EGFR inhibits Bim through the MAPK pathway and thus induces anoikis resistance in malignant tumors (54, 55). A study also revealed NF-κB as the downstream target of EGFR in the PI3K/AKT pathway and the inducer of anoikis resistance (56). Similarly, STAT3 was found to maintain FAK activation during detachment (57). Therefore, FAK might in turn promote anoikis resistance through the PI3K/AKT pathway. Apart from the above pathways, the amplification of EGFR also activates NF-κB through TMEM43/LUMA and results in anoikis resistance in glioma (58).

### IGFR

IGFR is a family of transmembrane receptor tyrosine kinases that includes IGF1R, IGF2R, insulin receptor (IR) and hybrid IR/IGF1R. IGFRs bind to their cognate ligands, namely, IGF1, IGF2 and insulin. Six IGF-binding proteins (IGFBP1-6) bind to and carry IGFs in serum. Then, they are cleaved by MMPs in focal adhesions in the ECM, and IGFs are released (59). Activated IGF1R is involved in cell growth and survival control and is crucial for tumor transformation and survival (59). A study showed that cell detachment enhances IGF1R signaling through the collagen IV/α2 integrin/FAK pathway and thus promotes anoikis resistance (60). IGF1R signaling is carried out through the PI3K-Akt pathway and MAPK pathway (61, 62). However, a further study noted that the protective effect of IGF1 against anoikis is transduced *via* the PI3K-Akt pathway but not the Grb2/Ras/MAPK pathway (63). Furthermore, IGF1R/PI3K/Akt signaling promotes anoikis resistance through activation of NF-κB (64).

IGFBP2 overexpression is common in high-grade glioma and is related to glioma progression (65). Moreover, IR and IGF1R recruit and activate STAT3 *via* RACK1 mediation and induce anoikis resistance (66). Thus, a theoretic autocrine feed-forward loop model is established in which STAT3 activation leads to the binding of nuclear IGFBP2 to IGF1, resulting in IGF1 secretion that activates IGF1R, which in turn activates STAT3 (67).

### Trk

Trks are a family of tyrosine kinases that regulate synaptic strength and plasticity in the nervous system (68). The Trk family has three members, each of which binds to different types of growth factors called neurotrophins (NTs). TrkA binds to nerve growth factor (NGF) and is also called a high affinity nerve growth factor receptor. TrkB binds to brain-derived neurotrophic factor (BDNF) and NT-4. TrkC binds to NT-3 (69). Upon ligand binding, Trks can activate effectors, including SHC1, FRS2, SH2B1, SH2B2 and PLCG1 (70). Through these effectors, the PI3K/AKT pathway, MAPK pathway and NF-κB are activated (71, 72).

Trks have been reported to affect tumor behavior. TrkA inhibits tumor growth, invasion and angiogenesis, while TrkB promotes anoikis resistance and metastasis (73). Studies have revealed that TrkB contributes to anoikis resistance through the above signaling pathway and EMT (74, 75). TrkB expression is upregulated in lower grade glioma, proneural GBM and methylated phenotype GBM, and TrkB-induced anoikis resistance has been confirmed in glioma (76, 77). Further study showed the participation of Zeb1, an E-cadherin repressor, in TrkB-induced anoikis resistance (78). Thus, TrkB promotes anoikis resistance through the PI3K/AKT pathway, MAPK pathway, NF-κB activation, EMT and E-cadherin inhibition.

### TGF-β

TGF-β is a multifunctional cytokine produced by white blood cells. Activated TGF-β forms a serine/threonine kinase complex and binds to TGF-β receptors (TGFBR1 and TGFBR 2) (79). Upon ligand binding, TGFRBR2 phosphorylates and activates TGFRBR1, which initiates a signaling cascade (80). In this way, TGF-β activates the SMAD pathway, which translocates the Smad complex into the nucleus to induce the transcription of different effectors. There are also non-Smad signaling pathways, including the MAPK and PI3K-AKT pathways, that are initiated in parallel and cooperate with the SMAD pathway (81). Through these pathways, TGF-β exerts both tumor-promoting effects and tumor-suppressing effects.

TGF-β signaling is highly active in high-grade gliomas, which exerts tumor-promoting effects on proliferation, angiogenesis and invasion (82). An *in vivo* study also proved that TGF-β promotes anoikis resistance upon glioma cell detachment (31). As the most potent inducer of EMT, TGF-β mainly promotes anoikis resistance through this transition and the subsequent loss of cell adhesion (83, 84). TGF-β also promotes anoikis resistance mediated by ROS and β-catenin (85, 86).

### The Hippo pathway

The Hippo pathway is a well-known signaling pathway that controls organ size by regulating cell proliferation and apoptosis (87). Large tumor suppressor (LATS) 1 and 2 are starting proteins of the Hippo pathway. LATS1/2 negatively regulate the transcriptional coactivator/corepressor YAP and TAZ. YAP/TAZ then bind to the transcription factor family TEAD and direct gene expression in the nucleus (88). The Hippo pathway is not directly activated by the loss of cell adhesion but by the signaling of other pathways (89). For example, YAP/TAZ are regulated by integrin-SRC signaling, E-cadherin-JUB signaling, growth factor-PI3K-Akt signaling and GPCR signaling (90). Thus, the Hippo pathway induces anoikis by transducing upstream signals.

Under physiological conditions, the Hippo pathway downregulates YAP and induces anoikis (91). However, it is one of the 8 signaling pathways that is frequently dysregulated in cancer (89). Evidence shows that YAP1 upregulation promotes anoikis resistance and EGFR amplification in GBM (92). YAP1 also regulates the glioma phenotype by inhibiting proneural marker OLIG2 expression (93). YAP knockdown increases apoptosis and attenuates GBM metastasis (94, 95). The YAP inhibitor verteporfin has been shown to have significant efficacy in a preclinical GBM model, indicating that the Hippo pathway is a promising therapeutic target for glioma (96).

## Cytoplasmic proteins and anoikis resistance

Cell detachment does not affect cytoplasmic proteins directly. However, proteins such as transcription factors and kinases can act as important mediators of anoikis resistance and are usually regulated by multiple pathways. Nuclear factor-κB (NF-κB), elongation factor-2 kinase (eEF-2 kinase) and Mps one binder kinases 2 (MOB2) are known promotors of glioma.

### NF-κB

NF-κB is a primary transcription factor that widely participates in cell proliferation and survival control (97, 98). NF-κB is a dimeric complex formed by RelA, RelB, NF-κB1, NF-κB2 and c-Rel. When bound to DNA, different dimer combinations act as transcriptional activators or repressors (99). NF-κB plays a key role in inducing anoikis resistance and is the downstream effector of many signal-transducing events. Inhibitors of κB (IκBs) block the nuclear entry of NF-κB and keep it in an inactive state. Activation of NF-κB is initiated by the signal-induced degradation of IκB proteins with the help of IκB kinase (IKK) (100).

NF-κB is dysregulated in most malignant tumors and causes aberrant expression of several anoikis-related proteins. The anoikis suppressors Bcl-2, BCL-xL, OPG, IAP, c-FLIP, and RIPK1 were upregulated by NF-κB *in vivo (* 101–103). NF-κB is also reported to counter reactive oxygen species (ROS)-induced anoikis through manganese superoxide dismutase (MnSOD) upregulation (104). High-grade and invasive gliomas often exhibit NF-κB dysregulation. NF-κB promotes the expression of ZEB-1 in glioma, which in turn represses E-cadherin and results in anoikis resistance (105). On the other hand, inhibition of the gene transactivation function of NF-κB by cannabidiol induces cell death in GBM (106).

### eEF-2 kinase

eEF-2 kinase is a calcium/calmodulin-dependent transferase. eEF-2 kinase phosphorylates eEF-2 and decreases its affinity for ribosomes, leading to retardation of protein elongation and therefore inhibition of protein synthesis (107). It was also discovered that eEF-2 kinase regulates dendritic mRNA translation in neurons (108). eEF-2 kinase is often upregulated in cancer cells and promotes proliferation, survival and migration (109).

eEF-2 kinase is upregulated in glioma (110). Upon detachment, the high expression of eEF-2 kinase induces anoikis resistance and promotes invasion through downregulation of Bim and upregulation of BCL-xL (111, 112). On the other hand, inhibition of eEF-2 kinase promotes anoikis and sensitivity to TMZ (113).

### MOB2

MOB2 regulates the activation of nuclear-Dbf2-related (NDR) kinases, which are associated with apoptosis signaling. MOB2 inhibits FAS-induced NDR activation and consequently suppresses apoptosis (114). *In vivo* and *in vitro* studies have shown that MOB2 is downregulated in GBM and that downregulation of MOB2 confers anoikis resistance (115). Further experiments revealed that anoikis resistance is achieved through the integrin/FAK/Akt pathway and the cAMP/PKA pathway (114, 115). Few studies have focused on MOB2 as a tumor suppressor, and its therapeutic value remains to be explored.

## The tumor microenvironment (TME) and anoikis resistance

The TME is the abnormal environment surrounding tumor cells. The TME has pathogenic properties such as hypoxia, acidosis and lack of nutrition. These properties and factors also regulate anoikis.

### Hypoxia and acidosis

Hypoxia is a standard property of the TME. Hypoxia-inducible factors (HIFs) are transcription factors that respond to hypoxia and are commonly upregulated in tumor cells. HIF promotes cell survival by upregulating several genes associated with angiogenesis and metastasis (116, 117). Therefore, it is natural for HIF to play a part in cancer cell survival. An *in vivo* study showed that HIF-1-induced anoikis resistance can be achieved by repression of the BH3-only proteins Bim and Bmf through the EGFR–Mek–Erk and AKT pathways (118, 119). HIF-1α also promotes anoikis resistance by upregulating ANGPTL4, which activates the FAK/Src/PI3K-Akt/ERK pathway and thereby suppresses Caspase activation (120). HIF-1α functions similarly and promotes anoikis resistance in glioma (121).

Acidosis is another property of the TME and is caused partially by hypoxia. Acidosis has dual effects on tumors: mild acidosis promotes survival, and severe acidosis suppresses survival. A study revealed that acidic pH promotes autocrine TGF-β2 signaling and in turn promotes anoikis resistance (122). *In vivo* studies also show that low pH induces anoikis resistance in melanoma (pH=6.7) and enhances proliferation in GBM (pH=6.2) (123, 124). However, a very low pH (pH=3.4) immensely increases the surface rigidity of GBM cells by altering the cholesterol and GM3 glycosphingolipid content and causes anoikis instead (124).

### ROS

ROS are highly reactive byproducts of cell metabolism (125). ROS are present at low and stable levels in normal cells and have roles in signaling and homeostasis (126, 127). However, under cancerous conditions, redox homeostasis is dysregulated, and ROS serve as a double-edged sword. Low levels of ROS facilitate cancer cell survival, while high levels of ROS and therapeutic-induced augmentation of ROS stress kill cancer cells (128, 129).

ROS play important roles in achieving anoikis resistance in cancer. An increase in ROS leads to constitutive inactivation of PTEN, which rescues PI3K-AKT signaling and integrin-mediated anoikis resistance (130). Sustained ROS levels also promote the activation of Src kinases and redox-sensitive transcription factors such as NF-κB and HIF-1α, thereby regulating cell adhesion and anoikis through degradation of Bim and upregulation of BCL-xL (130, 131). On the other hand, high levels of ROS promote anoikis sensitivity upon cell detachment, confirming the dual role of ROS in anoikis (132).

ROS function similarly in glioma. Prohibitin is upregulated in glioma stem-like cells (GSCs) and facilitates therapeutic resistance by regulating ROS (133). GBM cells with low Pax6 expression achieve anoikis resistance. Pax6-overexpressing cells retain higher levels of ROS upon detachment, and their survival is impaired (134). Treatments utilizing ROS stress as a mechanism have been successful. Celastrol induces ROS production and inhibits Akt and mTOR activity, thus triggering cell death in glioma (135). Dihydroartemisinin promotes ROS production, which activates p53 protein and in turn downregulates β-catenin expression, thus inhibiting glioma invasion and anoikis resistance (136).

### HSPs

HSPs are a family of proteins that respond to stress events and function as chaperones (137). HSPs are upregulated by heat shock factors (HSFs) during environmental stress conditions (138). The chaperone function of HSP helps stabilize partially unfolded proteins, assisting protein conformation establishment and preventing unwanted protein aggregation (139).

HSP upregulation is triggered in cancer by hypoxia, starvation and cancer therapy. Therefore, HSPs participate in cancer progression and therapy resistance. For example, HSP27 promotes astrocytoma migration through SPARC-MAPK signaling and is suppressed by PTEN (140). HSP70 and its cochaperone BAG3 induce anoikis resistance in GBM through Bcl-2 overexpression (141).

### Protective autophagy and anoikis resistance

Autophagy is a cellular process that promotes cell adaptation through the degradation of dysfunctional or unnecessary components. Generally, autophagy is triggered upon stress events such as starvation and infection, therefore promoting cell survival. However, autophagy in cancer can be a double-edged sword. While high-level autophagy has an antitumor effect, low-level autophagy can relieve cancer cells of the pressure from hypoxia, acidosis and lack of nutrients. It also facilitates the degradation of apoptotic mediators, thus interfering with apoptosis or anoikis (142). In short, autophagy can play a protective role in cancer and induce anoikis resistance upon cell detachment.

The mechanism of protective autophagy-induced anoikis resistance is not clear, as various involved pathways have been reported. One review concluded that protective autophagy is induced *via* integrin-focal adhesion signaling (143). Another *in vivo* study specified the ATF4/CEMIP/PKCα pathway as a trigger of protective autophagy (144). A study in glioma, however, revealed the MDA-9/Syntenin pathway as the key regulator in maintaining protective autophagy (145). These inducers of protective autophagy can be potential therapeutic targets. However, further investigation is required to fully understand the balance between protective autophagy and anoikis.

## Anoikis resistance and current glioma therapy

The common protocol for glioma contains surgery, radiotherapy, conventional chemotherapy, and target therapy (146). Part of these treatments involve anoikis mechanism and their efficacy are affected by anoikis resistance.

### Anoikis resistance and radiotherapy

Radiotherapy is a standard measure in glioma management, although gliomas often show high radioresistance. A study in prostate cancer shows that one of the reasons for radiotherapy resistance is the acquirement of anoikis resistance in detached tumor cells through Erk and PI3k-Akt signaling (147). Consistently, AKT inhibitor MK2206 is able to enhance the sensitivity of radiotherapy and inhibit invasion in glioma, which could result from the inhibition of anoikis resistance (148). A recent study identifies an adhesion molecule CD146 as a mediator of radiotherapy resistance through suppression of p53 expression and activation of NF-κB, which could induce anoikis resistance (149). Thus, it is highly possible that anoikis resistance takes part in the resistance of radiotherapy in glioma.

### Anoikis resistance and chemotherapy

Conventional chemotherapy for glioma includes procarbazine, lomustine, carmustine, vincristine, and temozolomide (146). Among them, TMZ is the most common adjuvant treatment. TMZ is an alkylating agent commonly used in gliomas, especially in astrocytoma and GBM. A recent study deliberately cultures anoikis-resistant astrocytes to form clones and test their resistance to TMZ. After treatment with 0.72mM TMZ, only one out of three of these astrocyte-derived clones show a significant decrease in cell viability, with slowed but not halted colony formation. The unsatisfying response to TMZ of these astrocytes is similar to that of the established GBM cell line U87MG (150). Therefore, anoikis resistance is likely one of the main reasons for TMZ resistance.

### Anoikis resistance and target therapy

Target therapies exceed conventional chemotherapy in preventing drug resistance and often generate a synergistic response. Therefore, target therapies aiming at the anoikis mechanism are an ideal adjuvant treatment for glioma. Target drugs relating to anoikis are concluded in **Table 1**.

**Table 1:** Anoikis related.drugs.

Drug	Mechanism	Design	Result	Conclusion	Reference
Cilengitide	Integrin inhibitor	Phase II clinical trial of cilengitide monotherapy in refractory pediatric glioma	One response out of 24 subjects median OS = 172 days	Cilengitide is not effective as a single agent for refractory pediatric high grade glioma	(151)
		Randomized Phase II clinical trial of cilengitide combined with Chemoradiation in GBM	Median OS of all groups = 19.7 months; median OS of 500mg cilengitide group = 17.4 months; median OS of 2000mg cilengitide group = 20.8 months	Cilengitide is well tolerated when combined with standard chemoradiation and may improve survival for patients newly diagnosed with GBM	(152)
		*In vivo* study of cilengitide with human pediatric glioma cell lines and U87MG cell line	/	Cilengitide is able to induce cell detachment and anoikis leading to critical growth inhibition in pediatric cells expressing αvβ3	(153)
		*In vivo* study of cilengitide and bevacizumab combine therapy with rat orthotopic glioma model	/	The combination of bevacizumab with cilengitide exert a significant anti-invasive effect	(154)
Gefitinib	EGFR inhibitor	Phase II clinical trial of gefitinib in recurrent GBM	Median OS = 39.4 weeks	Gefitinib is well tolerated and has activity in patients with recurrent glioblastoma	(155)
		Phase II clinical trial of gefitinib in grade 4 astrocytoma	12-month rate for OS of historical control population = 48.9%; 12-month rate for OS of gefitinib group = 54.2%; 12-month rate for PFS of gefitinib group = 16.7%; 12-month rate for PFS of historical control population = 30.3%	Treatment with adjuvant gefitinib post radiation was not associated with significant improvement in OS or PFS	(156)
		Phase II clinical trial of gefitinib and irradiation in pediatric brainstem glioma	Median FPS = 7.4 months	Administration of gefitinib with irradiation in children with brainstem glioma is well tolerated	(157)
		Phase I/II clinical trial of radiation therapy with concurrent gefitinib for GBM	Median PFS = 4.9 months; Median OS = 11.5 months	The addition of gefitinib to RT is well tolerated but has no significant improvement in Median OS	(158)
Erlotinib	EGFR inhibitor	Phase I/II clinical trial of bevacizumab, irinotecan and erlotinib in pediatric diffusive intrinsic pontine glioma	Median PFS = 7.3 months; Median OS = 13.8 months	Daily erlotinib is safe and well tolerated in doses up to 85 mg/m2 when combined with biweekly bevacizumab and irinotecan in children with progressive DIPG	(159)
OSI-906	InsR/IGF1R inhibitor	*In vivo* study of OSI-906 and gefitinib combine therapy with mice subcutaneous GBM model	/	OSI-906 synergistically improves GBM sensitivity to gefitini	(160)
Larotrectinib	Trk inhibitor	Phase I/II clinical trial of larotrectinib in TRK fusion-positive glioma	12-month rate for PFS = 56%; 12-month rate for OS = 85%	Larotrectinib demonstrated rapid and durable responses, high disease control rate, and a favorable safety profile in patients with TRK fusion-positive glioma	(161)
Trabedersen	TGF-β2 inhibitor	Phase IIb clinical trial of trabedersen in GBM and astrocytima	Median OS of GBM with 10μM trabedersen = 12.0 months; Median OS of GBM with chemotherapy = 10.1 months; Median OS of astrocytoma with 10μM trabedersen = 39.1 months; Median OS of astrocytoma with 80μM trabedersen = 35.2 months; Median OS of GBM with chemotherapy = 21.7 months;	10 mM trabedersen is the optimal dose for high-grade glioma. The 6-month intratumoral convection-enhanced delivery of trabedersen was found to be safe and well tolerated	(162
	TGF-β1 inhibitor	Phase Ib/IIa clinical trial of galunisertib, temozolomide and radiotherapy combined in glioma	Median OS of galunisertib + TMZ + radiotherapy group = 18.2 months;	No differences in efficacy, safety or pharmacokinetic variables were observed	(163)
		Phase II clinical trial of galunisertib monotherapy and lomustine monotherapy and combine therapy in GBM	Median OS of TMZ + radiotherapy group = 17.9 months Median OS of galunisertib group = 8.0 months; Median OS of galunisertib +lomustine group = 6.7 months; Median OS of placebo + lomustine group = 7.5 months	Galunisertib + lomustine failed to demonstrate improved OS relative to placebo + lomustine. Efficacy outcomes were similar in all 3 arms	(164)
Cediranib	VEGF inhibitor	*In vivo* study of cediranib with U87MG, U251MG and T98G GBM cell lines	/	Cediranib synergistically increased the anti-proliferative and apoptotic effects of radiotherapy and bevacizumab and augmented the sensitivity of GBM cells to temozolomide chemotherapy	(165)

Cilengitide, an integrin inhibitor, is able to induce dose-dependent cell detachment of pediatric glioma cells expressing αvβ3 and trigger anoikis. However, the adult glioma cell line U87MG is able to grow despite cilengitide-induced nonadherent conditions. These results indicate that cilengitide cannot reduce anoikis resistance (153). Consistent with this conclusion, only 1 out of 24 subjects has a response in phase 2 clinical trials of cilengitide treating high-grade pediatric gliomas (151). Despite the unsatisfying effect of cilengitide as monotherapy, combined therapy trials with radiation and TMZ, bevacizumab, or other agents have shown promising outcomes (152, 154, 166). Therefore, integrin inhibitors are still reliable adjuvant treatments for glioma treatment.

EGFR inhibitors such as erlotinib and gefitinib have been developed and applied in the clinical treatment of many other cancers. However, despite the common amplification of EGFR in GBM, EGFR inhibitors have been disappointing in single drug clinical trials (155, 156). Nevertheless, combined therapies have shown promising results. Combined treatment of erlotinib with bevacizumab and irinotecan has shown prolonged OS (Median OS = 13.8 months) in children with progressive diffuse intrinsic pontine glioma (159). Adjuvant gefitinib treatment also improved radiotherapy outcome in children (Median PFS =7.4 months) but not in adult (Median PFS = 4.9 months; Median OS = 11.5 months) (157, 158). Thus, EGFR inhibitors are recommended adjuvant treatments in pediatric glioma.

IGF1R inhibitor based treatment has not been successful, and multiple clinical trials of ganitumab have been halted due to lack of benefit (167). However, it is reported that the frequent activation of IR and IGF1R in GBM confers resistance to EGFR inhibitors, and IGF1R has a compensatory effect during EGFR inhibition (160, 168). These finding indicate a promising future for combined treatment with EGFR inhibitor and IGFR inhibitor.

TRK fusion leads to constitutive activation of Trk in all types of gliomas (169). The Trk inhibitors entrectinib and larotrectinib have been approved for the treatment of TRK-fusion glioma (170). While both drugs are still in trial, larotrectinib has demonstrated favorable efficacy in treating glioma (23 responses of 28 subjects; 12-month rate for PFS = 56%; 12-month rate for OS = 85%) (161). Therefore, Trk inhibitors are expected in the future management of TRK-fusion glioma.TGF-β pathway antagonist like trabedersen has been proposed for glioma treatment for a long time (82). A clinical trial of 145 patients shows that trabedersen improves OS in GBM (Median OS 12.0 months vs. 10.1 months) and astrocytoma (Median OS 39.1 months vs. 21.7 months) (162). Another antagonist galunisertib is also trialed in glioma but shows insignificant improvement (Median OS 18.2 months vs. 17.9 months) (Median OS 8.0 months vs. 7.5 months) (163, 164). Many other novel antagonists are developed for cancer treatment, and combined therapies are also expected in the future (171).

HSP is a promising therapeutic target in terms of both monotherapy and combined therapy. A clinical trial shows that immunotherapy targeting HSP96 improves OS in recurrent GBM (Median OS 47 weeks vs. 16 weeks) (172). And an *in vivo* study shows that silencing HSP27 and HSP72 improves the efficacy of TMZ in glioma (173).

Apart from therapies that target the anoikis mechanism, cediranib, a VEGF inhibitor also affects anoikis resistance in GBM. Study shows that cediranib reduce the survival of anoikis-resistant GBM cells, especially in the T98G cell line, while the U87MG cell line shows intermediate resistance. The study also reveals the synergy effect of cediranib with radiotherapy and TMZ in glioma treatment (165). There has been no clinical trial for cediranib in glioma up till now. However, anti-VEGF drugs like bevacizumab and regorafenib are already in the guideline for glioma management (146).

## Conclusion

Currently, anoikis resistance in glioma has not been extensively studied. Nevertheless, the diffuseness of glioma invasion and the high recurrence rate are closely linked to anoikis resistance. Therefore, clarification of the regulation of anoikis resistance at the molecular level could provide a new angle for glioma therapy and prognosis evaluation. In this article, we reviewed current publications on the role of adhesion molecules, signaling pathways, cytoplasmic molecules, the TME and protective autophagy in anoikis resistance and their therapeutic value in glioma. Further studies, however, are still needed to validate the function of these molecules in anoikis resistance and glioma. More research is necessary to explore new directions for targeting anoikis resistance in the treatment of glioma.

## Author contributions

ZZ, CF, and HX designed the review and wrote the manuscript. LY and YX conceived the artwork and performed the bibliographical research. AS, AZ, and ML supervised the writing. All the authors revised and approved the final version of the manuscript.

## Funding

This work was supported by the Natural science foundation of Shanghai (18ZR1430400) and the Zhejiang Provincial Natural Science Foundation of China (LY22H090020).

## Conflict of interest

The authors declare that the research was conducted in the absence of any commercial or financial relationships that could be construed as a potential conflict of interest.

## Publisher’s note

All claims expressed in this article are solely those of the authors and do not necessarily represent those of their affiliated organizations, or those of the publisher, the editors and the reviewers. Any product that may be evaluated in this article, or claim that may be made by its manufacturer, is not guaranteed or endorsed by the publisher.
